# The Nutritional Status of Individuals Adopted Internationally as Children: A Systematic Review

**DOI:** 10.3390/nu13010245

**Published:** 2021-01-16

**Authors:** Richard Ivey, Marko Kerac, Michael Quiring, Hang T. Dam, Susie Doig, Emily DeLacey

**Affiliations:** 1 Department of Population Health, Faculty of Epidemiology and Population Health, London School of Hygiene & Tropical Medicine, University of London, London WC1E 7HU, UK; marko.kerac@lshtm.ac.uk (M.K.); emilyd@holtinternational.org (E.D.); 2 Centre for Maternal, Adolescent, Reproductive & Child Health (MARCH), London School of Hygiene & Tropical Medicine, University of London, London WC1E 7HU, UK; 3 Holt International, Eugene, OR 97401, USA; michaelq@holtinternational.org (M.Q.); hangd@holtinternational.org (H.T.D.); susied@holtinternational.org (S.D.)

**Keywords:** international adoption, children, nutritional status, malnutrition, growth

## Abstract

Since 1955, international adoption has been a way of finding homes for children who have been orphaned or abandoned. We aimed to describe the nutritional status of individuals adopted internationally and their long-term nutritional and health outcomes. We searched four databases for articles published from January 1995 to June 2020, which included information on anthropometric or micronutrient status of children adopted internationally (CAI). Mean Z-scores on arrival to adoptive country ranged from −2.04 to −0.31 for weight for age; −0.94 to 0.39 for weight for height; −0.7 to 0 for body mass index; −1.89 to −0.03 for height for age; −1.43 to 0.80 for head circumference for age. Older children, those adopted from institutionalized care or with underlying disability, were more likely to be malnourished. Though long-term data was scarce, mean Z-scores post-adoption ranged from −0.59 to 0.53 for weight for age; −0.31 to 1.04 for weight for height; 0.39 to 1.04 for body mass index; −1.09 to 0.58 for height for age; −0.06 to 1.23 for head circumference for age. We conclude that though CAI are at high risk of malnutrition at baseline, marked catch-up growth is possible, including for those older than two years of age on arrival. This has implications not only for CAI but for the wider population of malnourished children worldwide. Research on how to optimize catch-up growth is a priority.

## 1. Introduction

Globally, there are some 140 million children worldwide who are orphans, defined as those aged younger than 18 who have lost “one or both parents to any cause of death” [1]. There are also an estimated 60 million children living on the streets worldwide and 10 million more living in institutions [2], which is defined by the United Nations as residential care that is provided in any non-family-based group setting [3,4].

UN international adoption began in 1955 as a response to post-World War II societal destruction and continues to be a method to find homes for children who have been orphaned or abandoned. Detailed statistics are difficult to find, but one 2007 review described “a silent global movement of about 30,000 children per year moving between about 100 different countries” [5]. Children often move from low- and middle-income countries to high-income countries, primarily the United States, Spain, France and Italy [6].

Adopted children are by definition vulnerable and often experience medical issues such as growth faltering and developmental delay related to their difficult early childhood and suboptimal pre-adoption quality of care [7]. International adoption aims to provide them with a safe, family-based environment, where the quality of care, attachment, interaction and nutrition experienced often improves [8]. The better-quality environment is crucial to improving the child’s health because many CAI have been legally relinquished for adoption by one or both birth parents (South Korea, Thailand, Ethiopia, etc.) who are still living, while others are removed from caregivers due to severe abuse and neglect, and, as a result, are at high risk of malnutrition at baseline on arrival to their adoptive country [9].

### 1.1. Malnutrition Epidemiology

In 2019, 144 million (21.3%) children younger than 5 years old worldwide were stunted (i.e., too short for their age, commonly interpreted as a marker of chronic undernutrition), 47 million (6.9%) were wasted (too thin, commonly interpreted as a marker of acute undernutrition), and 38.3 million were overweight or obese [10]. Undernutrition is particularly concerning short term since is it associated with 45% of deaths among children younger than 5 years old [10].

### 1.2. Malnutrition and Disability at Pre-Adoption Baseline

Many children who are adopted internationally arrive into their adoptive country with specific and sometimes extensive medical needs. These include underlying infectious disease, inadequate nutrition, histories of low birth weight and psychological deprivation [11,12,13]—all of which are associated with delayed growth and cognition [12,13]. Disabilities are also common, particularly among those who arrive from institutionalized care [14,15]. Since disability and malnutrition often intersect [16], those with disabilities can become even more at risk of malnutrition and malnutrition in turn can exacerbate and lead to new disabilities [16]. For example, 90% of children with cerebral palsy have difficulty feeding, which can lead to inadequate intake of nutrients [17].

### 1.3. Malnutrition Post-Adoption

There is currently little research describing the long-term health outcomes following international adoption. As well as being of direct relevance to adoptees, this is of interest to those working in severe malnutrition in resource-poor and humanitarian settings [18]. Whilst the traditional focus of treatment and prevention programs has been on averting short-term malnutrition-associated mortality, there is increasing realization of adverse long-term consequences including a higher risk of chronic diseases in adulthood [19,20,21,22]. This relates to the “thrifty phenotype” hypothesis which arises from observations that reduced fetal growth, caused by maternal malnutrition, is strongly associated with several chronic health conditions later in life [23]. In contrast, some other authors find that early growth restriction does not increase the risk of metabolic syndrome if they have a healthy post-natal nutritional environment [24]. Examining outcomes for CAI may shed further light on key factors and mechanisms which are also relevant to the wider population of children recovering from any type of early life malnutrition. This is because CAI typically relocate from rural to urban settings or from low-income countries with traditional diets to high-income countries with obesogenic environments and Western diets with excess calories, fats and carbohydrates [24]. This mirrors the similar but longer-term changes within all countries with increasing urbanization and dietary changes.

### 1.4. Research Gap

Though there have been two reviews to date synthesizing evidence on growth in CAI, both are now more than 10 years old and there is need for an update [25,26]. There is also need for a more direct approach: Mason and Narad, for example, focused on the underlying causes of delayed growth in CAI but had insufficient on-arrival and post-adoption anthropometric data, which limited their ability to describe catch-up growth [26].

This review aims to describe and understand the baseline and long-term nutritional (anthropometric and micronutrient) status of individuals who were adopted as children, with particular emphasis on understanding factors influencing that, namely:

- Pre-adoption factors, e.g., early life clinical and nutritional history; underlying disability.
- Peri-adoption factors, e.g., age at adoption; length of stay in any institutional care before adoption.
- Post-adoption factors, e.g., socioeconomic and nutritional environment into which children are adopted.

## 2. Materials and Methods

We conducted a systematic review with narrative synthesis to understand baseline (at adoption) and longer-term post-adoption nutritional status of children adopted internationally.

### 2.1. Protocol and Registration

We followed PRISMA guidelines [27] throughout the study (List S1) and completed PROSPERO registration prior to the start of the study (PROSPERO 2020: CRD42020186825 https://www.crd.york.ac.uk/prospero/display_record.php?RecordID=186825) [28].

### 2.2. Eligibility Criteria

We used the following PICO:

Population: Individuals who were adopted internationally as children (aged younger than 18 years).

Intervention: Our exposure was “international adoption”.

Comparator: Due to likely paucity of data on our target population, we considered all types of studies, including those with a comparator group.

Main outcomes: Nutritional status as assessed by:

- Anthropometric data, including weight for age, weight for height, height for age, head circumference for age and body mass index. Our main focus was on standardized values using WHO growth standards, but we also considered other growth references (e.g., CDC, NCHS growth references) and non-standard reports (e.g., unadjusted height or weight).
- Micronutrient status: either laboratory-measured values or clinical status if applicable (e.g., clinically obvious rickets suggesting vitamin D deficiency).

Studies were eligible if they met the following criteria:

- Peer-reviewed studies.
- Written in English.
- Include at least one measurement of nutritional status, either micronutrient status or anthropometric data through standardized tools, such as WHO Growth Standards [29] or Centers of Disease Control growth charts [30].
- Published from January 1995 to July 2020.

We chose studies published after 1995, which is both the baseline for the Millennium Development Goals and also when the Hague Convention on the Protection of Children and Co-operation in Respect of Intercountry Adoption entered into force. This is an international treaty that provides necessary safeguards to protect the best interests of children, birth parents and adoptive parents who are involved in intercountry adoptions, especially in respect to protecting children from corruption, abuses and exploitation [31].

Studies were excluded if:

- They reported on individuals adopted at the age of 18 years or older.
- They focused on domestic adoption placements.
- Study reports were not peer reviewed.
- They used non-standardized anthropometric growth measurements.

### 2.3. Information Sources and Search Strategy

We searched four electronic databases through OVID between 15 June and 30 June 2020: Medline, Embase, Global Health Database and CINHAL Plus. Lead researcher RI conducted the initial title/abstract screen. Uncertainties about which items to include in the final tables were discussed with other co-authors, with EDL making the final decision about any discordant records. The electronic search strategy used for Medline is attached in Supplementary Materials (List S2).

### 2.4. Data Extraction

We extracted data on gender, age of adoption, standardized anthropometric measurements, micronutrient status, disability status, country of birth and adoption and entered them into Excel tables.

### 2.5. Quality Assessment

The NICE quality assessment tool was used to measure the risk of bias of individual studies. There are five sections in this appraisal tool. Section 1 aims to assess external validity, while Section 2, Section 3 and Section 4 assess the critical criteria for determining the study’s internal validity [32]. The NICE quality assessment results can be found in Table 1 and greater detail in the Supplementary Material Table S2.

### 2.6. Summary Measures

We summarized the results by describing the range of mean on arrival and post-adoption anthropometric Z-scores. Ranges of the mean prevalence of malnutrition indicators and micronutrient deficiencies were recorded and compared with arrival and post-adoption data.

## 3. Results

### 3.1. Study Selection

Our search terms identified a total of 4939 papers. After 1518 duplicates were removed, 3421 papers were screened by title/abstract, of which 62 were potentially eligible. Of these 62, we were unable to locate the full text for four studies, which is partly because many journals do not have online access. A total of 12 studies had insufficient or non-standard anthropometric data and 6 included those not within our pre-determined population. This left us with a final 24 papers which met the inclusion criteria.

**Figure 1:**
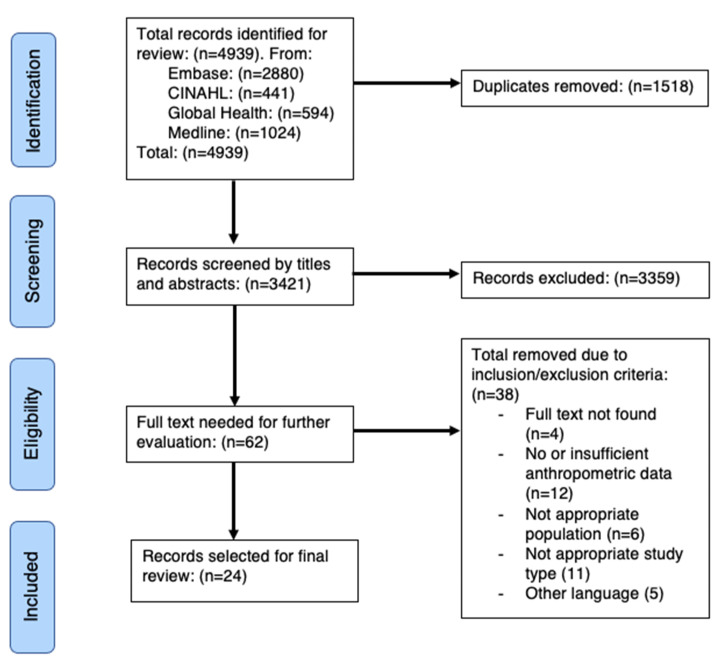
Flow diagram of study selection.

Our study flow chart is shown below in Figure 1. Studies excluded are listed in Table S1.

### 3.2. Study Characteristics

The 24 papers included were published between 1997 and 2019 and eight (33%) were published in the past five years. Studies included various countries to which children were adopted; 13 were to USA (54%), five were to Italy (20%), two were to Spain (8%), two were to Canada (8%) and two were to other European countries (8%). All were reports of observational studies, of which nine (37%) were cross-sectional studies and 13 (54%) were cohort studies. Anthropometric and micronutrient data was reported in three studies pre-adoption (12%), 20 on arrival (83%) and nine post-adoption (37%). On arrival anthropometric and micronutrient data was measured within one week to seven months after arrival.

### 3.3. Anthropometric Data

Eighteen studies (75%) in Table 2 reported anthropometric data using World Health Organization (WHO) growth standards/references, Centre of Disease Control (CDC) standards or North American norms. The prevalence of underweight, wasting, stunting, overweight and microcephaly was reported in six (25%), six (25%), nine (37%), one (4%) and six (25%) studies, respectively. One study reported weight and height measurements as percentiles of the WHO growth charts/references [35]. Of the 24 studies, three (12%) reported anthropometric information pre-adoption, 16 (66%) on arrival, and nine (37%) post-adoption.

### 3.4. Weight

On arrival to their adoptive country, the mean WAZ score ranged from −2.04 to −0.31, compared with −0.59 to 0.53 post-adoption (see Table 2). On arrival, WHZ score ranged from −0.94 to 0.39, compared to −0.31 to 1.04 post-adoption. One study reported 123 children (49%) weighted below the third percentile [35]. Prevalence of wasting ranged from 0% to 18.4% on arrival, which is almost three times greater than the global prevalence of 6.9% [10]. Post-adoption, for those adopted, Fuglestad et al. did not find adoptees to be wasted [33]. Studies reported BMI as BMI for age Z-score and the mean BMI. On arrival, BMI for age Z-scores ranged from −0.7 to 0, compared to 0.39 to 1.04 post-adoption. Mean BMI on arrival was 16 to 23.93, compared to 21.67 to 25.85 post-adoption. Johansson-Kark et al. reported an overweight prevalence of 8.4% to 28.6% for adults who were adopted internationally as children [45].

### 3.5. Height

Prevalence of stunting from arrival to post-adoption; 12% to 39%, compared to 17% post-adoption. On arrival, mean HAZ ranged from −1.89 to −0.03 and post-adoption HAZ ranged from −1.09 to 0.58. One study reported 40% of children were below the third percentile for height on arrival [35].

### 3.6. Head Circumference

On arrival, mean HCAZ ranged from −1.43 to 0.80 and post-adoption HCAZ ranged from −0.06 to 1.23. The prevalence of microcephaly (HCAZ < −2) at baseline ranged from 5% to 17%, compared to 4% post-adoption.

### 3.7. Micronutrient Status

Of the 24 studies reviewed, 10 (41%) reported micronutrient data. Table 3 shows that iron deficiency ranged from 15% to 25% at baseline. Fuglestad et al. found that children adopted from Eastern Europe to the USA had a 9 percentage point reduction in iron deficiency from arrival to 6 months; however, this reduction was determined to not be statistically significant [11]. The prevalence of anemia on arrival ranged from 9.6% to 54.4%. No studies reported the prevalence of anemia post-adoption. The prevalence of vitamin D deficiency on arrival ranged from 9% to 85.1% (Table 3). Three studies reported mild, moderate and severe vitamin D deficiency, of which the prevalence ranged from 32.1% to 33.6%; 38.4% to 40.5%; and 9.6% to 12.5%, respectively. Only one study reported a prevalence of vitamin D deficiency post-adoption which found no significant improvement from baseline [33]. Two studies reported information on rickets, with a prevalence of 15.4% and 100%; the latter study was specifically regarding three cases of rickets [43,52]. Fuglestad et al. reported that 68% of CAI had at least one abnormal nutritional biochemical marker on arrival to their adoptive country [33]. The most common deficiencies they reported were low retinol-binding protein (33%), zinc deficiency (29%), vitamin D insufficiency/deficiency (21%) and iron deficiency (15%).

### 3.8. Age of Adoption

On arrival, the mean age ranged from 11 months to 5.31 years. A total of 10 (41%) studies reported an association between age of adoption and anthropometric or micronutrient data. Four studies reported an inverse correlation between age of adoption and height [14,36,41,48]. Johnson et al. reported that HAZ was inversely associated with the age of adoption for those who previously lived in institutionalized care (*p* = 0.01), meanwhile, they found no association between HAZ and age of adoption for children who lived in foster care before adoption (*r* = −0.15, *N* = 26, ns) [48]. Miller et al. reported for children younger than two years at arrival, age at arrival was inversely associated with height (*p* < 0.01), weight (*p* < 0.01) and head circumference *(p* < 0.02), regardless of the location of residence before adoption [41]. Conversely, three studies found no association between age at adoption and delay in growth for either height, weight or head circumference on arrival to the adoptive country [9,48,50]. Pomerleau et al. found no significant association between age of adoption and growth indicators when they measured weight, height and head circumference six months post-adoption [37]. Interestingly, Palacios et al. reported no significant relationship between the age at adoption and anthropometric indicators three years post-adoption, except for head circumference (*r* = 0.13, *p* < 0.05) [36].

## 4. Discussion

### 4.1. Summary of Evidence

The study findings found that CAI were commonly malnourished when they arrived in their adoptive families. The majority of these children are affected by multiple forms of undernutrition and micronutrient deficiencies. The prevalence of undernutrition was comparable regardless of their country of origin and sex; however, CAI from institutionalized care had significantly more delays in weight, height and head circumference than those adopted from other care settings. Children adopted at an older age were also more malnourished than younger children, regardless of their prior living situation. There is some evidence on the nutritional status of CAI on arrival, but less is known about to what extent international adoption impacts long-term health and growth. In our review, only a few studies described the nutritional status of CAI post-adoption, but those that did suggest that substantial catch-up growth is possible for all CAI in weight, height and head circumference [11,33,36].

### 4.2. Pre-Adoption Factors, Country of Origin and Adoption

Data sources were limited, but there were no obvious patterns of anthropometric deficit varying by country of origin in most studies. Miller et al., however, found that children from Ethiopia/Eritrea had significantly better growth at arrival than international adoptees from China, Guatemala and Russia [50]. One study also reported post-adoption, the country of origin is significantly related to differences in weight and head circumference [36]. Only two studies reported information on nutritional status after three years of arrival, and both included anthropometric data on adults who were previously adopted internationally [42,45]. Both papers concluded that adulthood weight of individuals previously adopted internationally varied by the country of adoption [42,45]. There could many underlying reasons for this, including early life epigenetic changes or diversity in susceptibility to being overweight [54,55]. However, the limited available data means we are unable to quantify associations or infer causality and distinguish pre- and peri-adoption factors from post-adoption environments, diets and lifestyles. Future research measuring the nutritional status of adults who were adopted internationally as children is needed to understand the association between international adoption and risk of becoming overweight or obese in adulthood.

### 4.3. Micronutrient Status

Micronutrient deficiencies were prevalent in CAI on arrival, which is attributable to poor nutrition and infections [11]. The prevalence of anemia was similar to the global average for children younger than 5 years old, which is likely to be contributing to delayed linear growth and weight gain [56,57]. Multiple studies found no statistically significant difference in the prevalence of iron deficiency at arrival and follow up for all CAI (*p* = 0.37) [11,33]. Fuglestad et al. reported that this association was virtually unchanged when controlling for daily iron intake at baseline and iron intake did not predict changes in serum ferritin [11]. The insufficient change in iron deficiency may partly be due to the negative correlation between growth rate and change in serum ferritin concentrations between baseline and follow up (*p* < 0.05) [11]. Further, anemia was not tracked post-placement in any of the studies. Fuglestad et al. additionally reported no statistically significant difference in the prevalence of iron deficiency and zinc deficiency at baseline and at follow up [33]. Iron deficiency was associated with lower cognitive scores (*p* < 0.03) and slower speed of processing (*p* < 0.02); meanwhile, there was an association between zinc deficiency and compromised memory functioning (*p* < 0.01) [33]. The prevalence of micronutrient deficiencies did not improve significantly post-adoption, except for an increase in serum zinc concentrations (*p* < 0.01) [33].

Vitamin D deficiency was common for CAI, with the prevalence peaking at 53% on arrival. Only one (4%) study measured the prevalence of vitamin D deficiency on arrival and post-adoption [33], from which they reported vitamin D insufficiency was significantly higher in children from the post-Soviet states (*p* < 0.01) compared to Ethiopia and China. The variance may be because the children adopted from the post-Soviet states were from the northern-most latitude in their sample and spent the most time in institutionalized care, which limited their sun exposure and vitamin D synthesis. There was no significant difference in vitamin D deficiency at arrival and at follow up, despite participants being treated with 2000 IU vitamin D daily for eight weeks. Adoptees with a lower BMI and longer time in institutionalized care were more likely to be vitamin D deficient after adjusting for age [34]. CAI commonly live in institutionalized care before adoption; therefore, it is plausible to assume these children may have increased prevalence of vitamin D deficiency because of their lack of sun exposure or limited diets [34].

These results highlight the need to consider the possibility of micronutrient deficiencies in CAI and where appropriate formally measure micronutrient status. Interventions that provide nutritional supplements to young children can have both short-term and long-term benefits, such as treating anemia [58] and improved reading scores and non-verbal cognitive ability tests in adulthood [59].

### 4.4. Age at Adoption

The age at which children are adopted is also an important factor which may affect the nutritional status of CAI at baseline/on arrival and catch-up growth post-adoption. In our review, six papers reported an association between age of adoption and anthropometric data at baseline/on arrival [9,14,36,41,48,50]. This data is important to understand which peri-adoption factors contribute to delayed growth in CAI. Overall, the results in our review were inconsistent and contradict previous research, which found that the age of adoption was not significantly related to weight, height and head circumference Z-scores on arrival [25].

The age at which children are adopted, and begin to experience improved WASH practices, nutrition and psychological support could also affect catch-up growth in CAI. The first 1000 days of life are highlighted in current global health policy as offering a key window of opportunity for growth and development which will affect the health of individuals throughout their life [60]. In the short term, as much as 70% of linear growth deficit at 60 months is due to faltering during the first 1000 days [61]. The importance of growth and development in the first 1000 days is well established. However, children are often adopted internationally after this period. Therefore, previous studies have adjusted for age of adoption when analyzing the nutritional status of CAI to understand to what extent catch-up growth is possible after the child turns 2 years old. Children adopted at an older age experience longer exposure to negative risk factors such as inadequate nutrition and psychological deprivation, which may increase their growth delays, especially for children who lived in institutionalized care prior to international adoption [43].

In our review, the insufficient number of long-term studies describing catch-up growth in CAI limited our ability to describe the association between age of adoption and catch-up growth. Despite this, one study in our review that followed children for three years post-adoption found no significant association between age of adoption and catch-up growth for weight and height; however, they found a significant inverse association between age of adoption and head circumference catch up [36]. This association is probably due to the increased duration of psychosocial deprivation, which is associated with reduced head growth, even in the absence of subpar nutrition [62]. Our findings are in contrast with previous research which found that later age at arrival was associated with less complete catch up of height and weight [25,63]. The age at which children arrive to their adoptive family may also impact their health in the long term. For example, children adopted after the age of 2 have increased odds of becoming overweight in adulthood compared to those adopted before their first birthday [45].

### 4.5. Institutionalized Care and Orphanages

Similar to a previous review, we found limited data regarding the association between institutionalized care and growth indicators for CAI. The papers that did include this data support the existing literature that suggests that children who live in institutionalized care prior to being adopted internationally have significantly more delays in weight, height and head circumference on arrival to their adoptive country [25,26], particularly for those with a more extended stay in institutionalized care [43,46,53]. Linear growth faltering in these children reflects long-term chronic difficulties [64], which include inadequate nutrition, psychological care and impacts to growth hormones related to stressful early childhood experiences [65]. Children residing in orphanages who experience inadequate care have abnormal and high cortisol levels [65,66]. CAI have also demonstrated a relationship between the length of time spent in an orphanage and high cortisol levels when cortisol was measured 6.5 years after adoption [67]. Despite increased delays in growth on arrival and the potential long-term adverse health consequences, CAI who lived in institutionalized care prior to adoption demonstrate significant catch-up growth for weight, head circumference and height [36]. One study in our review reported that as much as 65% of children demonstrated catch-up growth (>0.5 change in Z-score) in length from arrival to follow up [11].

Although there was limited data, our review suggests that catch-up growth for children who have resided in institutionalized care is potentially comparable to that of all CAI. Future research with longer follow-up periods is needed to fully describe the post-adoption nutritional status of CAI from institutionalized care.

### 4.6. Sex and Nutrition Status

Sex is an essential factor to consider because females and males may not receive a similar quality of care before adoption, as well as the physiological differences between females and males [68]. In our review, no studies reported a significant association between sex and weight, height and head circumference growth on arrival. There was also limited anthropometric data post-adoption regarding differences in sex. This is an important area of international adoption research considering the growing evidence that suggests that CAI are at higher risk of developing precocious puberty [69]. Teilmann et al. followed CAI during 39,978 person-years at risk and reported 45 girls and six boys developed precocious puberty; girls adopted internationally had a 10 to 20 times greater risk of developing precocious puberty compared to girls who had a Danish background [69]. The earlier pubertal maturation has been hypothesized to be caused by the stressful psychosocial factors which adoptees experience during infancy. When central precocious puberty is not treated at an early stage, individuals can experience compromised final adult height [70]. Early menarche may also increase a person’s risk of metabolic syndromes such as adulthood obesity, cardiovascular disease and diabetes [71].

Notably, only one (4%) study measured the relationship between anthropometric data and sex, post-adoption and found that Korean-born adult males had consistently greater BMI than females (*p* < 0.01). However, this may have been related to high BMI in male adults who lived the USA compared to Europe [42]. Increased prevalence of micronutrient deficiencies was not associated with sex, except one study which found that females had a significantly worse vitamin D status (*p* < 0.05) [40]. However, they found no significant difference between mean 25-hydroxyvitamin D values in females and males (*p* = 0.59). Further analysis determined that sex was significantly associated with vitamin D status only when testing a severe versus a moderate vitamin D deficiency, with female children having an increased risk of developing severe vitamin D deficiency compared to male children (OR 0.55, 95% CI: 0.36–0.86) [40]. In contrast, Chiappini et al. found no association between sex and vitamin D status for children adopted to Italy [44].

### 4.7. Disability and Nutritional Status

There is a high prevalence of children with disabilities living in out-of-home care or available for adoption due to economic and social constraints faced by families in low-income countries [72]. Families that adopt children domestically also often request to be placed with children who are young and healthy, which contributes to the high prevalence of children with disabilities remaining in institutions available for international adoption [73]. These children are some of the most vulnerable children during the international adoption process due to their increased risk of malnutrition, which is attributable to their disability status and increased time living in institutionalized care [14,43]. In our review, only one paper measured the nutritional status of children with disabilities, which amplifies the need for more research to allow families to understand the needs of their child better.

### 4.8. Strengths and Limitations

This review has many strengths which improve the understanding of the nutritional status of CAI throughout all stages of the adoption process. This study supports previous reviews that found that the majority of CAI are at high risk of malnutrition on arrival, especially those who previously lived in institutionalized care and those adopted at an older age. Our review also found that all CAI can experience catch-up growth, despite their country of origin, sex and previous living situation. In contrast to previous reviews, we found significant improvements in head circumference post-adoption. We have also highlighted the potential association between the country of origin, the adoptive country and an increased risk of becoming overweight. However, gaps in the literature were also identified, such as the need to describe the long-term health impacts of international adoption, particularly longer than three years post-adoption. Research focusing on the nutritional status of adult adoptees will identify whether there is an increased risk of obesity and non-communicable disease, which may become apparent. This would support earlier intervention for health care providers or adoption agencies to provide targeted education and preventative health services for CAI and potentially reduce their risk of non-communicable diseases.

The lack of research is probably due to the practical issues of conducting long cohort studies, and challenges of collecting quality data without biases such as recall bias. Lost to follow up is also a common issue within international adoption cohort studies. One study in our review reported that post-adoption anthropometric data was missing for 21% of their participants [33]. Inconsistent or inadequate anthropometry resulted in limited comparison of pre-adoption and post-adoption status. Several studies also failed to capture the information collected as part of the initial medical exam for the adoptees but allowed for subsequent follow-up medical records to be used instead; for example, in one study, the “on arrival” anthropometric data was measured from 1 week to 17 months after arrival. As a result, some children may have experienced catch-up growth prior to their initial screening, which reduces the comparability of anthropometric data. Additionally, there are many factors which may contribute to delayed growth in childhood such as small for gestational age, preterm birth, care practices, poor maternal health, poor feeding practices, low birth weight, infectious diseases, disability status and psychological deprivation [74,75,76,77]. Often there is minimal information available to researchers about children’s early life. Hence, there are many biases inherent to the studies that we found, summarized by the NICE quality assessment in Table 1. We also acknowledge that we may have missed some papers due to single screening of titles and abstracts by our lead author rather than double screening by two or more authors. Our review also only included research published in English from January 1995 to July 2020, excluding other potentially relevant studies that were published in other languages or that were conducted outside of the identified timeframe. Given the limitations and biases inherent to almost all studies we did find, we feel it very unlikely that other papers would have changed our overall conclusions—what is needed to move the science forward in this field is not more of the same but better designed, higher-quality new research.

A key finding from our review was the limited amount of long-term studies measuring the nutritional status of CAI post-adoption. This radical change of environment and the impact to children’s nutritional status have very important implications and additional research should also explore the impact in other migrant groups of children. Prospectively following CAI and measuring their nutritional status (anthropometric and micronutrient status) from baseline up to years after adoption will help to inform more of the impacts of environmental shifts. Important variables to focus this research on will include the impact of sex, institutionalized care, age of adoption, disability, country of origin, country of adoption and maternal health on catch-up growth. Addressing the numerous research gaps will support caregivers, health care providers and families to better understand the needs of CAI to support optimal growth and development.

## 5. Conclusions

The number of international adoptions has dramatically reduced over the last decade, and there is an increasing number of in-country adoptions, which is encouraging. However, in-country adoption is not possible for every child and, therefore, international adoption continues to be necessary to ensure every child has the opportunity to grow up in a safe, loving family. Based on the research of the nutritional status of CAI on arrival to their adoptive country, it is evident that CAI have a high risk of being malnourished on arrival to their adoptive country, especially those coming from institutionalized care, those with disabilities and those adopted at an older age. Encouragingly, we found evidence to suggest that the radical change of environment can result in substantial catch up in weight, height and head circumference. While the first 1000 days are important, our results found that CAI can experience catch-up growth even when adopted in their later years of childhood. Every child has the right to thrive nutritionally and adoption gives valuable lessons in the fight against malnutrition.

## Figures and Tables

**Table 1:** Description of studies included in the review.

Author, Year	Study Design	Country	Study Population	NICE Quality Assessment Score: Internal Validity/ External Validity Score	Timing of Nutritional Assessments	Sex
Fuglestad et al., 2016 [33]	Prospective cohort	Multi country 🡲USA	*N*: 58 children. Participants included children aged 8–18 months.	+/+	Arrival and 6 months post-adoption	Females: 32 (55%)
Gustafson, Eckerie et al., 2013 [34]	Prospective cohort	Multi country 🡲USA	*N*: 160 patients. Aged 4 months to 17.8 years.	+/−	Within 6 months of adoption	Females: 83 (52%)
Park, Bothe et al., 2011 [9]	Prospective cohort	Multi country 🡲USA	*N*: 58 children. Mean age on arrival: 17.6 months. Children evaluated within 19 days.	−/−	On arrival	Females: 34 (58%)
Bortone, Totaro et al., 2019 [14]	Prospective cohort	Multi country 🡲Italy	*N*: 422 children. Median age at arrival: 6.5 years. Adopted from Europe (29.9%), Asia (26.8%), Africa (23.9%) and Latin America (19.4%).	+/−	Median 75 days after arrival	Females: 171 (40.5%)
Fuglestad et al., 2008 [11]	Prospective cohort	Multi country 🡲USA	*N*: 37 children. Children adopted from orphanages or hospitals. Low birth weight: 32%.	−/−	On arrival and 6 months post-adoption	Females: 22 (59%)
Martinez Ortiz, Dominguez Pinilla et al., 2015 [35]	Retrospective cohort	Ethiopia 🡲Spain	*N*: 251 children. Mean age of arrival: 7 months. 124 (49.4%) aged ≤ 6 months.	−/+	Pre-adoption	
Palacios, Roman et al., 2011 [36]	Retrospective cohort	Multi country 🡲Spain	*N*: 289 children. Mean age on arrival: 34.9 months. Children from institutionalized care. Parents reported the results (approximately 38 months after arrival) of their child’s on arrival medical tests.	+/+	On arrival and 3 years post-adoption	
Pomerleau et al., 2005 [37]	Prospective cohort	China, Vietnam, Taiwan, Thailand, South Korea, Cambodia, Russia and Belarus 🡲Canada	*N*: 123 evaluated. All children were adopted before 18 months of age. Adopted from orphanage, family-setting and some children had experience in both living situations. Assessed within one month of arrival (mean: 19.1 days).	−/−	On arrival, 3 months post-adoption and 6 months post-adoption	Females: 87 (70%)
Le Mare and Audet, 2006 [38]	Prospective cohort	Romania 🡲Canada	*N*: 36 evaluated. Lived in an orphanage for a minimum of nine months (9 to 53 months, mean = 24 months). Mean age at arrival: 23.9 months. Mean time in institution: 22.7 months.	+/−	11 months post-adoption, 4.5 years of age and 10.5 years of age	Females: 19 (53%)
Buonsenso, Graffeo et al., 2019 [39]	Retrospective cohort	Multi country 🡲Italy	*N*: 584 evaluated, (82.19%) lived in institutions. Mean age at arrival: 5 years and 9 months.	+/+	On arrival	
Salerno, Ceccarelli et al., 2018 [40]	Retrospective cohort	Multi country 🡲Italy	*N*: 873 children. Children were adopted from Europe and Russian federation (256, 29.8%), Latin America (231, 26.9%), Asia and Indian subcontinent (223, 26.0%), and Africa (149, 17.3%). Mean duration of institutionalization: 3 years.	+/+	On arrival	Females: 376 (43.8%)
Miller, Chan et al., 2005 [41]	Retrospective cohort	Guatemala 🡲USA	*N*: 103 children. Mean age on arrival: 16 months. Before adoption, 25 children resided in orphanages, 56 in foster care, and 22 in mixed-care settings (time living with birth family, foster care and orphanage).	+/+	On arrival	Females: 48 (47%)
Ulijaszek and Schwekendiek 2013 [42]	Retrospective cohort	Korea 🡲United States and Europe	Mean age when evaluated: 28.65 to 31.87 years old. Children adopted to America (52%) and Western Europe (44%). Adults self-reported their weight and height.	+/−	Post-adoption (mean age: males 30.46 years, females 28.65)	Females: 172 (66%)
Cataldo and Viviano 2007 [43]	Cross sectional	Multi country 🡲Italy	*N*: 36 children. Mean age at arrival: 78.5 months. Referred within 2–6 weeks of arrival.	−/−	Pre-adoption medical records and on arrival	Females: 62 (46%)
Chiappini, Vierucci et al., 2016 [44]	Cross sectional	Multi country 🡲Italy	Median age at arrival: 5.47 years 962 adopted from Africa (18.09%), South America (21.41%), Asia (16.32%), Europe (44.18%).	+/−	Median 72 days after arrival	Females: 381 (39.60%)
Johansson-Kark, Rasmussen et al., 2002 [45]	Cross sectional	Multi country 🡲Sweden	275,026 were included in study. 2400 adults who were international adoptees were evaluated. Mean age at adoption was 1.7 years. 64% adopted before age 2.	+/+	Post-adoption (17 years old)	Males: 275,026 (100%)
Miller and Hendrie 2000 [46]	Cross sectional	China 🡲USA	*N*: 452 children. The clinic group age at arrival: 2 months to 12 years and 4 months. Age at clinic visit: 3 months to 151 months. Children evaluated within 1.3 months.	−/+	1 week to 17 months of arrival	Females: 443 (98%)
Van Kesteren and Wojciechowski 2017 [47]	Retrospective study	Ethiopia 🡲Belgium	*N*: 315 children. Mean age on arrival: 3 years old.	+/+	On arrival	Females: 151 (48%)
Johnson, Bruce et al., 2011 [48]	Cross sectional	Multi country 🡲USA	*N*: 120 children. Mean age on arrival: 6.85 years. Three groups: Post-institutionalized children, children from foster care and non-adopted children raised in the US.	+/−	On arrival	All three groups had: Male: 10 Female: 30
Miller, Spratt et al., 2015 [49]	Cross sectional	Russia 🡲USA	*N*: 60 children. Age ranged from 3–10 years old. Three groups of children: previously institutionalized international adoptees, children with a history of neglect born in the USA, and controls.	−/+	Post-adoption (mean age 6.1 years old)	
Miller, Tseng et al., 2008 [50]	Cross sectional	Ethiopia/Eritrea 🡲USA	*N*: 50 children. 62% were less than 4 years old. Mean age on arrival: 3 months to 15 years. Mean age at clinic visit: 51.12 months.	−/+	On arrival	
Tirella and Miller 2011 [51]	Cross sectional	Multi country 🡲USA	*N*: 387 children. Mean age on arrival: 14.0 months. 86% of the children were evaluated within 2 months and 91% evaluated within 5 months of arrival.	−/−	On arrival	Females: 254 (66%)
Reeves, Bachrach et al., 2000 [52]	Case study	Soviet Union 🡲USA	Case 1: Age 2 years and 5 months, Case 2: Age 3 years and 3 months, Case 3: Age 2 years and 10 months.	−/+	On arrival	Females: 2 (66%)
Albers, Johnson et al., 1997 [53]	Case study	Russia🡲USA	*N*: 56 adoptees from East Europe. Median age at arrival: 26 months.	+/+	Pre-adoption medical records and on arrival	Females: 30 (54%)

**Table 2:** Anthropometric measurements and results.

Author, Year	Growth Reference	Weight for Age (WAZ)	Weight for Length/Height (WHZ)	Length/Height for Age (HAZ)	Body Mass Index (BMI) for Age	Head Circumference for Age (HCAZ)	Other Observations
Albers, Johnson et al., 1997 [53]	WHO growth standards	Prevalence of WAZ pre-adoption <−1: 44% Mean WAZ baseline: −1.05 (SD ± 1.06) (range −3.15 to 1.26)		Prevalence of HAZ < −1 pre-adoption: 68% Mean HAZ on arrival: −1.41 (SD ± 1.37) (range −4.52 to 1.79)		Prevalence of HCAZ < −1 pre-adoption: 43% Mean HCAZ on arrival: −1.25 (SD + 1.00) (range, −3.7 to 0.62)	Growth delay in height (68%). Growth delay in head circumference (43%). Delay in linear growth directly correlated with the amount of time living in an orphanage (*p* < 0.001).
Bortone, Totaro et al., 2019 [14]	WHO growth standards	Underweight prevalence baseline: 13.2%. Mean WAZ baseline: −0.4, −0.6 (above age 5)	Wasting prevalence baseline: 4.3%. Mean WHZ: −0.5, −0.9 (above age 5)	Stunting prevalence baseline: 12.9%			Stunting common in children < 5 years and in those with a disability. Disability in 72/422 (17.1%).
Cataldo and Viviano 2007 [43]	WHO growth standards	Mean WAZ baseline: −0.97 (−3.97 to 2.27)	Wasting prevalence baseline: 18.4%	Mean HAZ baseline: −1.30 (–5.98 to 2.17) Stunting prevalence baseline: 19.1%		Mean HCAZ baseline: −0.58 (−2.1 to 3.3) HCAZ <−2: 8.8%	Total Iron deficiency anemia: 74. Total rickets: 21. Total delayed bone age: 17.
Fuglestad, Kroupina et al., 2016 [33]	WHO growth standards	Post-Soviet States: Mean WAZ Baseline: −0.31 (SD 1.05) 6 months follow up: 0.23 (SD 0.87)	Mean WHZ Baseline: 0.39 (SD 1.01) 6 months follow up: 0.66 (SD 1.04)	Mean WHZ Baseline: 0.39 (SD 1.01) 6 months follow up: 0.66 (SD 1.04)		Mean HCAZ Baseline: 0.05 (SD 1.31) 6 months follow up: 0.31 (SD 1.03)	Nutritional deficiencies were not eliminated at follow up. Significant growth improvements from baseline to follow up in HAZ, (*p* < 0.001), WAZ, (*p* < 0.001), WHZ, (*p* < 0.001), and OFCZ (*p* < 0.001).
		Ethiopia: Mean WAZ Baseline: −0.89 (SD 0.93). 6 months follow up: 0.26 (SD 0.91)	Mean WHZ Baseline: 0.17 (SD 0.84) 6 months follow up: 1.04 (SD 1.10)	Mean HAZ Baseline: −1.89 (SD 1.34) 6 months follow up: −1.09 (SD 1.23)		Mean HCAZ Baseline: 0.20 (SD 1.15) 6 months follow up: 1.23 (SD 1.14)	
		Mean WAZ Baseline: −0.56 (SD 0.80) 6 months follow up: 0.02 (SD 0.96)	Mean WHZ Baseline: −0.14 (SD 0.84) 6 months follow up: 0.39 (SD 1.01)	Mean HAZ Baseline: −0.93 (SD 1.30) 6 months follow up: 0.58 (SD 0.96)		Mean HCAZ Baseline: −0.37 (SD 0.92) 6 months follow up: −0.06 (SD 1.17)	
Fuglestad, Lehmann et al., 2008 [11]	Centers for Disease Control (CDC), 2000	Mean WAZ Baseline: −1.73 6 months follow up: 0.53	Mean WHZ Baseline: −0.63 6 months follow up: −0.02 (WHZ)	Mean HAZ Baseline: −1.24 6 months follow up: −0.49		Mean HCAZ Baseline: −0.67 6 months follow up: 0.11	Mean serum ferritin concentration lower than the US population at follow up. Children with giardia lamblia at baseline had worse iron status at baseline and follow up.
Johansson-Kark, Rasmussen et al., 2002 [45]	WHO growth standards				BMI range: 20.68 to 23.93 Overweight prevalence: 8.8–28.6% Overweight prevalence: 14.1% for non-adopted participants		1.36 (1.03–1.80) increase in the odds of becoming overweight in adulthood for those who arrived in their adoptive country at a young age (0–1 year old) compared to those adopted after the age of 2.
Martinez Ortiz, Dominguez Pinilla et al., 2015 [35]	WHO growth standards	49% of children <3rd percentile for weight at baseline		40% of children <3rd percentile for height at baseline			151 (65%) had malnutrition (details not specified). Low weight for height was related to age at adoption.
Miller and Hendrie 2000 [46]	WHO growth standards	Weight: −3.77 to −2.4 (mean, SD: −1.17, 1.00)	Wasting prevalence baseline: 18%	Height: −8.64 to −2.9 (mean, SD: −1.51, 1.4), 39% stunted		Microcephaly prevalence: 28%	The amount of time living in a orphanage in months was proportional to the linear growth lag (*r* = 0.90; *p* = 0.0001) for 192 Chinese adoptees. For every 2.86 months of stay in an orphanage, children lost 1 month of height age.
Palacios, Roman et al., 2011 [36]	WHO growth standards 1995	Mean WAZ Baseline: −1.48 Follow up: 0.09 Difference: (*p* < 0.001)		Mean HAZ Baseline: −1.46 Follow up: −0.1 Difference: (*p* < 0.001)		Mean HCAZ Baseline: −0.71 Follow up: −0.46 Difference: (*p* < 0.001)	No significant relationship between length of institutionalization or age at arrival and growth indicators. A longer stay in orphanages was related with greater height delays (*p* < 0.05). Less than 7 months in orphanage, there was a negative relationship between orphanage duration and head circumference (*p* < 0.05).
Park, Bothe et al., 2011 [9]	WHO growth standards 2006 and CDC 2000 (for those older than 5)	Underweight prevalence baseline: 10% Mean WAZ baseline: −1.4	Wasting prevalence baseline: 28% Mean WHZ baseline: −0.5	Stunting prevalence baseline: 17% Mean HAZ baseline: −1.1		Microcephaly prevalence baseline: 16% Mean HCAZ baseline: −0.8	Growth Z-scores less than zero at baseline: HCAZ (77%), HAZ (79%), WHZ (64%) and WAZ (90%). No significant relationship between age of participants at baseline and all growth Z-scores.
Salerno, Ceccarelli et al., 2018 [40]	WHO growth standards				Mean BMI baseline: 16		No significant difference between 25(OH)D mean values for the different BMI groups (*p* = 0.47).
Ulijaszek and Schwekendiek 2013 [42]	WHO growth standards				Adult BMI: USA: Males: mean BMI 25.85. Females: mean BMI 22.18. Europeans: Males: mean BMI 22.77. Females: mean BMI 21.67. USA over 25 BMI = 25.6% Europe over 25 BMI = 14.3%		Males had greater BMI than females (*p* < 0.001). Adoptees in Europe had lower BMI than those in the US (*p* < 0.001).
Van Kesteren and Wojciechowski 2017 [47]	WHO growth standards		Wasting prevalence baseline: 8.6%	Stunting prevalence baseline: 28.9% Severe stunting prevalence baseline: 11%			Microcephaly was uncommon. Moderate microcephaly in 8 (3.3%) children. Severe microcephaly in 2 (0.8%) children.
Johnson, Bruce et al., 2011 [48]	CDC, 2000	Mean WAZ baseline: −2.04 (post-institutionalized). Mean WAZ baseline: −0.23 (foster care).	Mean WHZ baseline: −0.94 (post-institutionalized) Mean WHZ baseline: −0.35 (foster care)	Mean HAZ baseline: −1.54 (post-institutionalized) Mean HAZ baseline: −0.03 (foster care)			For CAI linear growth delay was related with greater DSA and a more dysregulated diurnal cortisol rhythm.
Miller, Spratt et al., 2015 [49]	CDC, 2000	Mean WAZ post-adoption: −0.59	Mean WHZ post-adoption: −0.31	Mean HAZ post-adoption: −0.5			Three groups recruited: previously institutionalized CAI, US born children with history of neglect and control. Mean height growth was different (*p* < 0.05). Head circumference was significantly smaller (*p* < 0.05) in CAI.
Miller, Tseng et al., 2008 [50]	CDC, 2000	Mean WAZ baseline: −0.59 Underweight prevalence baseline: 8%		Mean HAZ baseline: −0.64 Stunting prevalence baseline: 12%		Microcephaly prevalence baseline: 6% Mean HCAZ baseline: −0.09	WHZ increased with age at adoption. Growth measurement Z-scores not related with age at arrival. Children from Ethiopia/Eritrea had significantly better anthropometric status at arrival than adoptees from China, Guatemala, or Russia.
Miller, Chan et al., 2005 [41]	CDC, 2000	Mean WAZ baseline: −1.0 Underweight prevalence baseline: 20%		Mean HAZ baseline: −1.04 Stunting prevalence baseline: 16%		Microcephaly prevalence baseline: 17% Mean HCAZ baseline: −1.08	Children who resided in orphanages had significantly lower Z-scores for all height, weight and head circumference. Children younger than 2 years at arrival, Z-scores for growth measurements related inversely with age at arrival.
Tirella and Miller 2011 [51]	CDC, 2000	Mean WAZ baseline: −1.17 Underweight prevalence baseline: 27%		Mean HAZ baseline −0.74 Stunting prevalence baseline: 13%		Microcephaly prevalence baseline: 14% Mean HCAZ baseline: 0.8	Children from Guatemala had greater delays in height (*p* = 0.007) and head circumference (*p* = 0.01) than those from the other countries, although these results are not significant after the Bonferroni correction.
Pomerleau et al., 2005 [37]	North American norms, 1979 (ANOVA: time, group)	Mean weight percentile baseline: 61.55 (*p* < 0.001)	Mean weight/height percentile baseline: 14.43 (*p* < 0.001)	Mean height percentile baseline: 8.44 (*p* < 0.001) Mean height/age percentile baseline: 7.35 (*p* < 0.001)		Mean head circumference percentile: 12.26 (*p* < 0.001)	On arrival, children from East Asia had higher percentiles for weight and height than Chinese or Russian children. Age at arrival was significantly associated with weight/height, height/age, head circumference percentile and weight percentile on arrival growth. Age at arrival was not associated with any growth indicators 6 months post-adoption.
Le Mare and Audet, 2006 [38]	CDC, 2006	Mean weight percentile (11 months post-adoption): 7.85 Mean weight percentile (4.5 years of age): 43.6 Mean weight percentile (10.5 years of age): 59.9		Mean height percentile (4.5 years of age): 36.98 Mean height percentile (10.5 years of age): 48.8			By phase 2 (4.5 years of age), children demonstrated almost complete weight catch up with only 3 (8.6%) children below the third percentile. By phase 3 (10.5 years of age), only 1 (2.8%) child had a weight score below the fifth percentile.

**Table 3:** Micronutrient status and clinical signs results.

Author, Year	Country	Micronutrient Status (On Arrival/Pre-Adoption)	Micronutrient Status (Post-Adoption)	Clinical Signs
Bortone, Totaro et al., 2019 [14]	Multi country 🡲Italy	Total vitamin D deficiency: 188/416 (45.2%).		Total anemia: 40/417 (9.6%). Anemia not a risk factor for stunting (*p* = 0.285).
Buonsenso, Graffeo et al., 2019 [39]	Multi country 🡲Italy	Total vitamin D deficiency: Moderate: 224 (38.4%) to mild: 196 (33.6%). Intestinal parasitic infections associated with vitamin D deficiency (*p* < 0.05).		
Cataldo and Viviano 2007 [43]	Multi country 🡲Italy			Total anemia: 74 (54.4%). Total rickets: 21 (15.4%).
Chiappini, Vierucci et al., 2016 [44]	Multi country 🡲Italy	Median 25(OH)D level: 22.0 ng/mL. 73.8% of had hypovitaminosis D. Children >6 years old had an adjusted odds ratio of vitamin D deficiency and hypovitaminosis 1.87 (*p* < 0.01) and 2.50 (*p* < 0.01) times higher than children <6 years old. Age at arrival to Italy was significantly associated with both with 25-hydroxyvitamin D mean values (*p* < 0.01) and Vitamin D status (*p* < 0.01). Sex, country of origin and BMI-z-score < −2 were not associated with vitamin D status.		
Fuglestad, Kroupina et al., 2016 [33]	Multi country 🡲USA	Low retinol-binding protein (33%). Zinc deficiency (29%). Vitamin D insufficiency/deficiency (21%). Iron deficiency (15%).	No significant change in micronutrient at baseline and follow up.	
Gustafson, Eckerie et al., 2013 [34]	Multi country 🡲USA	Total vitamin D deficiency: 7%. Total vitamin D insufficiency: 27%.		
Park, Bothe et al., 2011 [9]	Multi country 🡲USA	Total anemia: 6 (11.5%).		
Reeves, Bachrach et al., 2000 [52]	Soviet Union 🡲USA	Total vitamin D deficiency: 3 (100%).		Total rickets: 3 (100%).
Salerno, Ceccarelli et al., 2018 [40]	Multi country 🡲Italy	A statistically significant difference was found for skin color (*p* = 0.011), season at first blood draw (*p* < 0.001), the age at the first blood draw (*p* < 0.001) and Vitamin D status. Time from the arrival to initial evaluation was not significantly related with 25(OH)D mean values (*p* = 0.388) and Vitamin D Status (*p* = 0.912). Female children had increased risk of severe vitamin D deficiency.		
Fuglestad, Lehmann et al., 2008 [11]	Multi country 🡲USA	Total iron deficiency at baseline: 25%. Children with giardia lamblia had worse iron status at baseline and follow up. Growth rate was negatively related with change in serum ferritin concentrations between baseline and follow up (*p* < 0.05).	Total iron deficiency at follow up: 16%.	
Miller, Chan et al., 2005 [41]	Guatemala 🡲USA			Total anemia: 30%.

## Data Availability

The data presented in this study are available in article or supplementary materials.

## Supplementary Materials

The following are available online at https://www.mdpi.com/2072-6643/13/1/245/s1, List S1: Preferred Reporting items for Systematic Reviews and Meta-analysis (PRISMA) checklist; List S2: Literature search strategy used for electronic databases; Table S1: Studies excluded and reasons; Table S2: NICE quality assessment.

## Abbreviation

BMI	Body mass index
BMIZ	Body mass index Z-score
CAI	Children adopted internationally
CDC	Centers of Disease Control
HAZ	Height-for-age Z-score
HCAZ	Head circumference-for-age Z-score
LBW	Low birth weight
MDI	Mental scale index
OFCZ	Occipitofrontal circumference Z-score
PDI	Psychomotor development index
VDD	Vitamin D deficiency
WAZ	Weight-for-age Z-score
WHO	World Health Organization
WHZ	Weight-for-height Z-score
