# Supplementary material for: The Nutritional Status of Individuals Adopted Internationally as Children: A Systematic Review
Source: Nutrients. 2021 Jan 16;13(1):245. doi: 10.3390/nu13010245 (PMC7829835; doi:10.3390/nu13010245)
Supplement: Supplementary file 1 [file nutrients-13-00245-s001.pdf]

nutrients

Supplementary Material

The Nutritional Status of Individuals Adopted Internationally as Children: A Systematic Review

Richard Ivey [1,2,*], Marko Kerac [1,2], Michael Quiring [3], Dam Thi Thuy Hang [3], Susie Doig [3], Emily DeLacey [1,2,3]

[1] Department of Population Health, Faculty of Epide and nd ology and Population Health, London School of Hygiene & Tropical Medicine, University of London, London, UK; marko.kerac@lshtm.ac.uk (M.K.); emilyd@holtinternational.org (E.D.)
[2] Centre for Maternal, Adolescent, Reproductive & Child Health (MARCH), London School of Hygiene & Tropical Medicine, University of London, London, UK
[3] Holt International, Eugene, OR, USA; michaelq@holtinternational.org (M.Q.); hangd@holtvn.org.vn (D.T.T.H.); susied@holtinternational.org (S.D.)
* Correspondence: Richard.Ivey1@alumni.lshtm.ac.uk

List S1. Preferred Reporting items for Systematic Reviews and Meta-analysis (PRISMA) checklist

Section/topic	#	Checklist item	Reported on page #
**TITLE**			
Title	1	Identify the report as a systematic review, meta-analysis, or both.	1
**ABSTRACT**			
Structured summary	2	Provide a structured summary including, as applicable: background; objectives; data sources; study eligibility criteria, participants, and interventions; study appraisal and synthesis methods; results; limitations; conclusions and implications of key findings; systematic review registration number.	1
**INTRODUCTION**			
Rationale	3	Describe the rationale for the review in the context of what is already known.	3
Objectives	4	Provide an explicit statement of questions being addressed with reference to participants, interventions, comparisons, outcomes, and study design (PICOS).	4
**METHODS**			
Protocol and registration	5	Indicate if a review protocol exists, if and where it can be accessed (e.g., Web address), and, if available, provide registration information including registration number.	4
Eligibility criteria	6	Specify study characteristics (e.g., PICOS, length of follow-up) and report characteristics (e.g., years considered, language, publication status) used as criteria for eligibility, giving rationale.	4
Information sources	7	Describe all information sources (e.g., databases with dates of coverage, contact with study authors to identify additional studies) in the search and date last searched.	5
Search	8	Present full electronic search strategy for at least one database, including any limits used, such that it could be repeated.	5
Study selection	9	State the process for selecting studies (i.e., screening, eligibility, included in systematic review, and, if applicable, included in the meta-analysis).	5

Data collection process	10	Describe method of data extraction from reports (e.g., piloted forms, independently, in duplicate) and any processes for obtaining and confirming data from investigators.	5
Data items	11	List and define all variables for which data were sought (e.g., PICOS, funding sources) and any assumptions and simplifications made.	4
Risk of bias in individual studies	12	Describe methods used for assessing risk of bias of individual studies (including specification of whether this was done at the study or outcome level), and how this information is to be used in any data synthesis.	5
Summary measures	13	State the principal summary measures (e.g., risk ratio, difference in means).	5
Synthesis of results	14	Describe the methods of handling data and combining results of studies, if done, including measures of consistency (e.g., I^2) for each meta-analysis.	5
Risk of bias across studies	15	Specify any assessment of risk of bias that may affect the cumulative evidence (e.g., publication bias, selective reporting within studies).	5
Additional analyses	16	Describe methods of additional analyses (e.g., sensitivity or subgroup analyses, meta-regression), if done, indicating which were pre-specified.	5
**RESULTS**			
Study selection	17	Give numbers of studies screened, assessed for eligibility, and included in the review, with reasons for exclusions at each stage, ideally with a flow diagram.	6
Study characteristics	18	For each study, present characteristics for which data were extracted (e.g., study size, PICOS, follow-up period) and provide the citations.	8
Risk of bias within studies	19	Present data on risk of bias of each study and, if available, any outcome level assessment (see item 12).	5
Results of individual studies	20	For all outcomes considered (benefits or harms), present, for each study: (a) simple summary data for each intervention group (b) effect estimates and confidence intervals, ideally with a forest plot.	12
Synthesis of results	21	Present results of each meta-analysis done, including confidence intervals and measures of consistency.	12
Risk of bias across studies	22	Present results of any assessment of risk of bias across studies (see Item 15).	5
Additional analysis	23	Give results of additional analyses, if done (e.g., sensitivity or subgroup analyses, meta-regression [see Item 16]).	12

**DISCUSSION**			
Summary of evidence	24	Summarize the main findings including the strength of evidence for each main outcome; consider their relevance to key groups (e.g., healthcare providers, users, and policy makers).	25
Limitations	25	Discuss limitations at study and outcome level (e.g., risk of bias), and at review-level (e.g., incomplete retrieval of identified research, reporting bias).	29
Conclusions	26	Provide a general interpretation of the results in the context of other evidence, and implications for future research.	30
**FUNDING**			
Funding	27	Describe sources of funding for the systematic review and other support (e.g., supply of data); role of funders for the systematic review.	30

List S2. Literature search strategy used for electronic databases.

Databases: Medline, CINAHL PLUS, Global Health Database, EMBASE
Search strategy: Initial screening based on title and abstract, full text assessment to see if it matches inclusion/exclusion criteria and a data extraction table to summarise information on chosen articles. PRISMA guidelines.
Search terms:
1 AND 2 OR 3 will be used during the search process
1) International Adoption: adopt* AND (migration OR migrate* OR inter-country)
2) Nutrition: nutrition* OR malnutrition
3) Anthropometry: (“length-for-age” OR LFA OR LAZ OR linear growth OR stunt* OR wasting OR wasted OR oedematous OR edematous OR kwashiorkor OR protein-energy OR (SAM OR MAM OR GAM) OR “weight-for-length” OR WFL OR WLZ OR muac OR mid upper arm circumference OR underweight OR thinness OR “weight-for-age” OR WFA OR WAZ OR An?emi* OR H?emoglobin Level* OR BMI OR Body mass index OR overweight)
Inclusion criteria: Studies after 1995, English, contained research related to international adoption, children, nutrition, anthropometric data or micronutrient status, data based on those who were adopted internationally as children (under 18), peer reviewed and contains anthropometric data using standardized tools such as WHO growth standards.
Exclusion criteria: National adoption, insufficient anthropometric measurements, incorrect study population, no full text.
Medline:
Database: Ovid MEDLINE(R) <1946 to June Week 1 2020>
Search Strategy:
----
1 Adoption/ (4783)
2 Child, Adopted/ (78)
3 adopt*.mp. [mp=title, abstract, original title, name of substance word, subject heading word, floating sub-heading word, keyword heading word, organism supplementary concept word, protocol supplementary concept word, rare disease supplementary concept word, unique identifier, synonyms] (203773)
4 1 or 2 or 3 (203773) [TOTAL FOR ADOPTION]
5 exp Human Migration/ (26222)
6 exp "emigrants and immigrants"/ or "transients and migrants"/ (23421)
7 (migrant* or migration or immigrant* or transient* or international*).mp. (880486)
8 5 or 6 or 7 (888801) [TOTAL FOR international]
9 4 and 8 (14989) [TOTAL FOR INTERNATIONAL ADOPTION]
10 exp "nutritional and metabolic diseases"/ (1270864)
11 (nutrition* or malnutrition*).mp. (359644)
12 10 or 11 (1530028) [TOTAL FOR NUTRITION OR MALNUTRITION]
13 ("length-for-age" or LFA or LAZ or linear growth or stunt* or wasting or wasted or oedematous or edematous or kwashiorkor or protein-energy or SAM or MAM or GAM or "weight-for-length" or WFL or WLZ or muac or mid upper arm circumference or underweight or thinness or "weight-for-age" or WFA or WAZ or An?emi* or H?emoglobin Level* or BMI or Body mass index or overweight).mp. (535982)
14 exp Growth Disorders/ (32778)
15 body mass index/ (125696)
16 exp body weight changes/ or exp overweight/ or thinness/ (264293)

17 exp Anemia/ (160467)
18 13 or 14 or 15 or 16 or 17 (746420) [TOTAL FOR GROWTH DISORDERS ETC]
19 9 and 12 (901) [TOTAL FOR INT-ADOPTION AND (NUTRITION OR MALNUTRITION)]
20 9 and 18 (475) [TOTAL FOR INT-ADOPTION AND GROWTH DISORDERS]
21 9 and 12 and 18 (257) [TOTAL FOR INT-ADOPTION AND (NUTRITION OR MALNUTRITION) AND GROWTH DISORDERS]

19 or 20 will be used for the search.

Embase, Global Health and CINAHL PLUS:

1 Adoption/
2 limit 1 to yr="1995 -Current"
3 Child, Adopted/
4 limit 3 to yr="1995 -Current"
5 Adopt*.mp. [mp=title, abstract, heading word, drug trade name, original title, device manufacturer, drug manufacturer, device trade name, keyword, floating subheading word, candidate term word]
6 limit 5 to yr="1995 -Current"
7 2 or 4 or 6
8 exp Human Migration/
9 limit 8 to yr="1995 -Current"
10 exp "Emigrants and Immigrants"/
11 limit 10 to yr="1995 -Current"
12 (migrant* or migration or immigrant* or transient* or international*).mp. [mp=title, abstract, heading word, drug trade name, original title, device manufacturer, drug manufacturer, device trade name, keyword, floating subheading word, candidate term word]
13 limit 12 to yr="1995 -Current"
14 9 or 11 or 13
15 7 and 14
16 exp "nutritional and metabolic diseases"/
17 limit 16 to yr="1995 -Current"
18 (nutrition* or malnutrition*).mp. [mp=title, abstract, heading word, drug trade name, original title, device manufacturer, drug manufacturer, device trade name, keyword, floating subheading word, candidate term word]
19 limit 18 to yr="1995 -Current"
20 7 or 19
21 "length-for-age" or LFA or LAZ or linear growth or stunt* or wasting or wasted or oedematous or edematous or kwashiorkor or protein-energy or SAM or MAM or GAM or "weight-for-length" or WFL or WLZ or muac or mid upper arm circumference or underweight or thinness or "weight-for-age" or WFA or WAZ or An?emi* or H?emoglobin Level* or BMI or Body mass index or overweight).mp. [mp=title, abstract, heading word, drug trade name, original title, device manufacturer, drug manufacturer, device trade name, keyword, floating subheading word, candidate term word]
22 limit 21 to yr="1995 -Current"
23 exp Growth Disorders/
24 limit 23 to yr="1995 -Current"

25 exp Body Mass Index/
26 limit 25 to yr="1995 -Current"
27 body weight changes/ or exp overweight/ or thinness/
28 limit 27 to yr="1995 -Current"
29 exp body weight changes/ or exp overweight/ or exp thinness/
30 limit 29 to yr="1995 -Current"
31 exp Anemia/
32 limit 31 to yr="1995 -Current"
33 22 or 24 or 26 or 28 or 30 or 32
34 15 and 20
35 15 and 33
36 15 and 20 and 33
37 34 or 35

Table S1. Studies excluded and reasons

	Author, Year	Reason for exclusion
1	Balding C, 2015	Non-standard or insufficient Anthropometric measurements or micronutrient data
2	Baron S, 2000	Not appropriate population
3	Bureau J, 1999	Not appropriate population
4	De Martino M, 2019	Non-standard or insufficient Anthropometric measurements or micronutrient data
5	Diamond G, 2003	Not appropriate population
6	Jenista, 2001	Non-standard or insufficient Anthropometric measurements or micronutrient data
7	McGuinness T, 2006	Non-standard or insufficient Anthropometric measurements or micronutrient data
8	Miller B, 2009	Non-standard or insufficient Anthropometric measurements or micronutrient data
9	Mitchell M, 1997	Non-standard or insufficient Anthropometric measurements or micronutrient data
10	Albers, 2005	Non-standard or insufficient Anthropometric measurements or micronutrient data
11	Cataldo, 2006	Non-standard or insufficient Anthropometric measurements or micronutrient data
12	Cohen, 2008	Non-standard or insufficient Anthropometric measurements or micronutrient data
13	Olivan Gonzalvo G, 2018	Not appropriate population
14	Seminara S, 2011	Not appropriate population
15	Seneckyl Y, 2003	Non-standard or insufficient Anthropometric measurements or micronutrient data
16	Ijzendoorn M, 2007	Not appropriate study type
17	Mason P, 2002	Not appropriate study type
18	Mason P, 2005	Not appropriate study type
19	Mason P, 2005	Not appropriate study type

20	Barratt M, 2013	Not appropriate study type
21	Altemeier, 2000	Not appropriate study type
22	Schulte E, 2005	Not appropriate study type
23	Aita, 2011	Not appropriate study type
24	Cataldo F, 2012	Other language
25	Brown M. S, 1999	Could not find full text
26	Coulot, 2003	Other language
27	Johnson, 2000	Other language
28	Laubjerg M, 2006	Other language
29	Le Masme A, 1999	Other language
30	Miller, 1999	Not appropriate study type
31	Montano, 2013	Could not find full text
32	Nicholson, 2002	No anthropometric data
33	Paricio Talayero, 1998	Not appropriate population
34	Sonego, 2002	Other language
35	Tomlinson-Hansen S, 2015	No anthropometric data
36	Virdis R, 2019	Not appropriate study type
37	Virdis R, 2013	Could not find full text
38	Zaffaroni M, 2018	Could not find full text

Table S2. NICE risk of bias assessment

	Rating System
Best	++
	+
Worse	–
	NR (not reported)
	NA (not applicable)

	Population			Method of allocation to intervention (or comparison)										Outcomes						Analyses						Summary	
Paper	1.1	1.2	1.3	2.1	2.2	2.3	2.4	2.5	2.6	2.7	2.8	2.9	2.10	3.1	3.2	3.3	3.4	3.5	3.6	4.1	4.2	4.3	4.4	4.5	4.6	5.1	5.2
Albers, 1997	+	+	+	+	++	+	-	+	NA	+	-	++	++	++	+	++	++	++	+	+	NA	NA	NR	-	NR	+	+
Bortone, 2019	++	++	++	++	NA	NA	-	+	NA	++	+	NA	NA	++	++	++	++	++	+	NA	-	NA	NA	-	-	+	-
Buonsenso, 2019	++	++	++	+	++	++	-	-	++	++	++	NA	NA	++	++	+	++	-	-	+	NR	NR	++	++	++	+	+
Cataldo, 2007	++	++	-	-	++	++	-	+	NA	++	NA	NA	NA	++	+	++	++	NA	-	-	NA	-	+	-	-	-	-
Chiappini, 2016	++	++	+	++	+	NA	-	-	+	NA	++	NA	NA	++	++	++	++	NA	-	+	NA	NA	+	NA	-	+	-
Fuglestad, 2008	+	-	-	-	+	-	-	-	NA	++	++	NA	NA	++	-	+	++	NA	-	-	NA	-	-	++	-	-	-
Fuglestad, 2016	++	+	-	+	++	-	+	+	NA	+	++	NA	NA	++	-	+	++	+	-	-	NA	-	+	++	++	+	+

Gustafson, 2013	++	++	++	++	NA	NA	-	+	NA	++	+	NA	NA	++	++	++	++	++	+	NA	NA	-	-	-	-	+	-
Johnasson, 2002	++	++	-	+	+	+	+	+	+	++	+	NA	NA	++	+	+	+	+	+	+	NA	+	++	+	++	+	+
Johnson, 2011	++	++	-	-	++	++	+	+	+	+	+	NA	NA	++	-	+	++	-	-	-	-	+	+	-	-	+	-
Le Mare, 2006	++	-	-	++	++	-	-	+	+	+	++	NA	NA	-	-	-	++	+	++	+	NR	-	+	++	-	+	-
Ortiz, 2015	-	++	-	-	-	+	-	++	++	+	++	NA	NA	++	+	+	++	+	-	-	NA	+	-	++	+	-	+
Miller, 2015	-	++	-	-	++	+	-	+	+	+	NA	NA	NA	++	++	-	++	NA	-	-	NA	-	+	-	++	-	+
Miller, 2005	++	++	++	+	-	+	-	+	++	+	+	NA	NA	++	++	+	++	-	-	-	NA	-	+	++	++	+	+
Miller, 2008	+	-	-	+	+	++	-	-	++	++	NA	NA	NA	++	++	-	++	-	-	-	-	-	+	++	++	-	+
Miller, 2000	++	++	+	-	-	++	-	-	+	-	++	NA	NA	++	+	++	++	NA	-	-	NA	+	+	++	++	-	+
Palacios, 2011	++	++	-	-	+	+	-	+	+	+	NR	NA	NA	++	+	-	++	-	-	+	NR	-	+	++	+	+	+
Park, 2011	++	+	-	-	-	++	-	-	++	+	-	NA	NA	++	-	++	++	-	-	-	-	-	+	++	-	-	-
Pomerleau, 2005	++	++	-	+	++	++	-	+	++	+	-	NA	NA	++	+	++	++	++	-	-	NR	-	+	-	+	-	-
Reeves, 2000	++	-	-	-	++	++	+	+	++	+	++	NA	NA	++	+	+	++	++	+	-	NA	+	+	+	-	-	+

Salerno, 2018	++	++	+	+	+	++	+	+	++	++	NA	NA	NA	++	+	+	++	NA	-	++	NA	+	-	++	++	+	+
Ulijaszek, 2013	++	++	-	+	++	+	+	++	++	+	++	NA	NA	+	+	-	+	-	++	-	NA	NR	+	-	+	+	-
Tirella, 2011	++	++	-	-	++	+	+	++	++	+	NA	NA	NA	++	+	++	++	NA	-	-	NA	-	+	-	++	-	-
Van Kesteren, 2017	++	++	-	+	+	+	+	+	++	+	++	NA	NA	++	++	++	++	+	-	+	NA	-	++	++	-	+	+
